# Employment

**Published:** 1855-01

**Authors:** 


### EMPLOYMENT.

Employment! Employment!
O that is enjoyment!
There's nothing like "something to do;'
Good heart-occupation
Is health and salvation,
A secret that's known to but few.

Ye listless and lazy!
Ye heavy and hazy!
Give hearts, hands and feet full employment
Your spirits 'twill cheer up,
Your foggy brains clear up,
And teach you the real enjoyment.

The lilies they toil not,
They drudge not and moil not,
And yet they are cared for 'tis true;
But the lily, in beauty,
Fulfills its whole duty—
E'en lilies have something to do.

"They sew not, they spin not."
'Tis true—but they *sin* not;
They work, uncomplaining, God's will—
Their work not hasting,
Their time never wasting,
The laws of their nature fulfil.

Ye hands, white as lilies,
Remember God's will is,
*Who so shall not work shall not eat;*
'Tis heart-occupation
Prevents heart-starvation;
Would'st thou the great Lawgiver cheat?

Then up, man and woman!
But godlike—be human!
To self and to nature be true.
Employment! Employment!
O that is enjoyment!
There's nothing like "something to do."

—*The Beach Bird.*

